# Exploring psychosocial factors that influence smartphone dependency among Korean adolescents

**DOI:** 10.1371/journal.pone.0232968

**Published:** 2020-05-13

**Authors:** Hyeon Sik Chu, Young Ran Tak, Hanyi Lee

**Affiliations:** School of Nursing, Hanyang University, Seoul, South Korea; University of Sao Paulo Medical School, BRAZIL

## Abstract

This study investigated the relationships among psychosocial factors that contribute to smartphone dependency among South Korean adolescents. This cross-sectional study involved the secondary data analysis of the 2016 Korean Children and Youth Panel Survey, a nationwide multistage cluster survey. Data were collected from 1,840 7th grade students in South Korea and analyzed with descriptive statistics, Pearson’s correlation coefficients, and a path analysis using SPSS 21.0 and AMOS 23.0. The path analysis showed that self-esteem and aggressiveness directly influenced smartphone dependency, while affective parenting attitude, peer attachment, resilience, self-esteem, and depressive symptoms indirectly influenced it. The explanatory variables accounted for 18.3% of the total variance. In conclusion, parents’ education on positive parenting and guidance concerning adolescents’ smartphone use is necessary to reduce adolescents’ smartphone dependency. It may also prove effective to promote adolescents’ interpersonal skills and self-esteem to foster positive peer relationships and self-control concerning smartphone use.

## Introduction

Smartphones can be used for multiple purposes, including obtaining information from the Internet, participating in new forms of communication, and playing games. Smartphone use is not restricted by time and space, and this greatly improves users’ daily efficiency [1]. Smartphones have thus emerged as a key communication tool for adults and adolescents.

The smartphone ownership rate is increasing worldwide. Globally, it is highest in South Korea (94%) and about 95% of South Korean adolescents own a smartphone [2]. Smartphones are rapidly replacing personal computers; ownership increases users’ accessibility to the Internet and social media [3]. The appropriate use of smartphones has a positive impact on the development of self-identity, sociality, and creativity among adolescents [4]. However, excessive smartphone use exposes users to addiction, cyber-bullying, harmful Internet content, reduced physical activity, musculoskeletal diseases [1,5], and changes in the brain structure and functioning [6]. These factors offset the establishment of identity and negatively affect adolescents’ transition to adulthood. Consequently, preventative interventions that address these relationships have been discussed [7].

Prior research concerning the negative impact of smartphones has been based on the concept of “addiction.” However, this is problematic because it assumes that all smartphone users are potential addicts. Thus, the concept of “overdependence,” rather than “addiction,” has recently been adopted in research concerning excessive smartphones use [8].

Previous research revealed that 7^th^ grade students were at the highest risk for overdependence among Korean adolescents [9]; therefore, it is necessary to establish preventive interventions that focus on early adolescents and the factors that may affect their smartphone dependence, including psychological, peer and familial factors. Since parents play a primary role in adolescents’ socialization, parental attitude is critical in the development of psychological problems among adolescents, including depressive symptoms and aggressiveness [10]. Parental attitude has also been noted to be associated with smartphone dependency [11]. Adolescents who do not form a secure relationship with their parents tend to immerse themselves in virtual interpersonal relationships (and vice versa) [11,12].

As adolescents spend an increasing amount of time with their friends to be psychologically independent from their parents, peer attachment levels increase and peer relationships play an important role in psychological health, such as self-esteem and resilience [13]. For adolescents, smartphones serve as a link to maintaining social relationships in peer groups, which makes them more dependent on smartphones than other age groups. Adolescents who are smartphone dependent are likely to be socially isolated and focus instead on virtual social relationships, increasing their risk of cyber-delinquency [12]. Smartphone-dependent adolescents are also greatly affected by psychological factors. Psychological maladjustment, such as depressive symptoms and aggressiveness, is more likely to emerge as emotional and behavioral problems in adolescence [7,14]. Adolescent depressive symptoms and aggressiveness are factors that can predict smartphone dependency, therefore, smartphone can be used to avoid negative emotions as well as to experience positive emotions [6].

The formation of positive self-esteem and resilience in adolescence has been reported to serve as a protective factor against psychological problems and smartphone dependence by controlling for the effects of stress [13,15]. Adolescents with low self-esteem and low resilience tend to avoid “real life” stress and fulfill their social desires in a virtual space, thus leading to smartphone dependency [15].

However, previous research has mostly examined the relationship between parental attitude and smartphone dependency or the relationship between adolescents’ psychological problems and smartphone dependency [5,13,15]. Research that comprehensively examines these variables in specific social contexts to identify their underlying structural relationship is insufficient. In addition, existing research has focused on adolescents’ personality traits rather than variables that can mediate smartphone dependency, which has limited the development of interventions. Therefore, in this study, we identified factors that affect smartphone dependency and examined their direct and indirect effects on adolescents in the 7th grade—a time in which the proportion of smartphone-dependent adolescents is high.

### Conceptual framework

**Fig 1:**
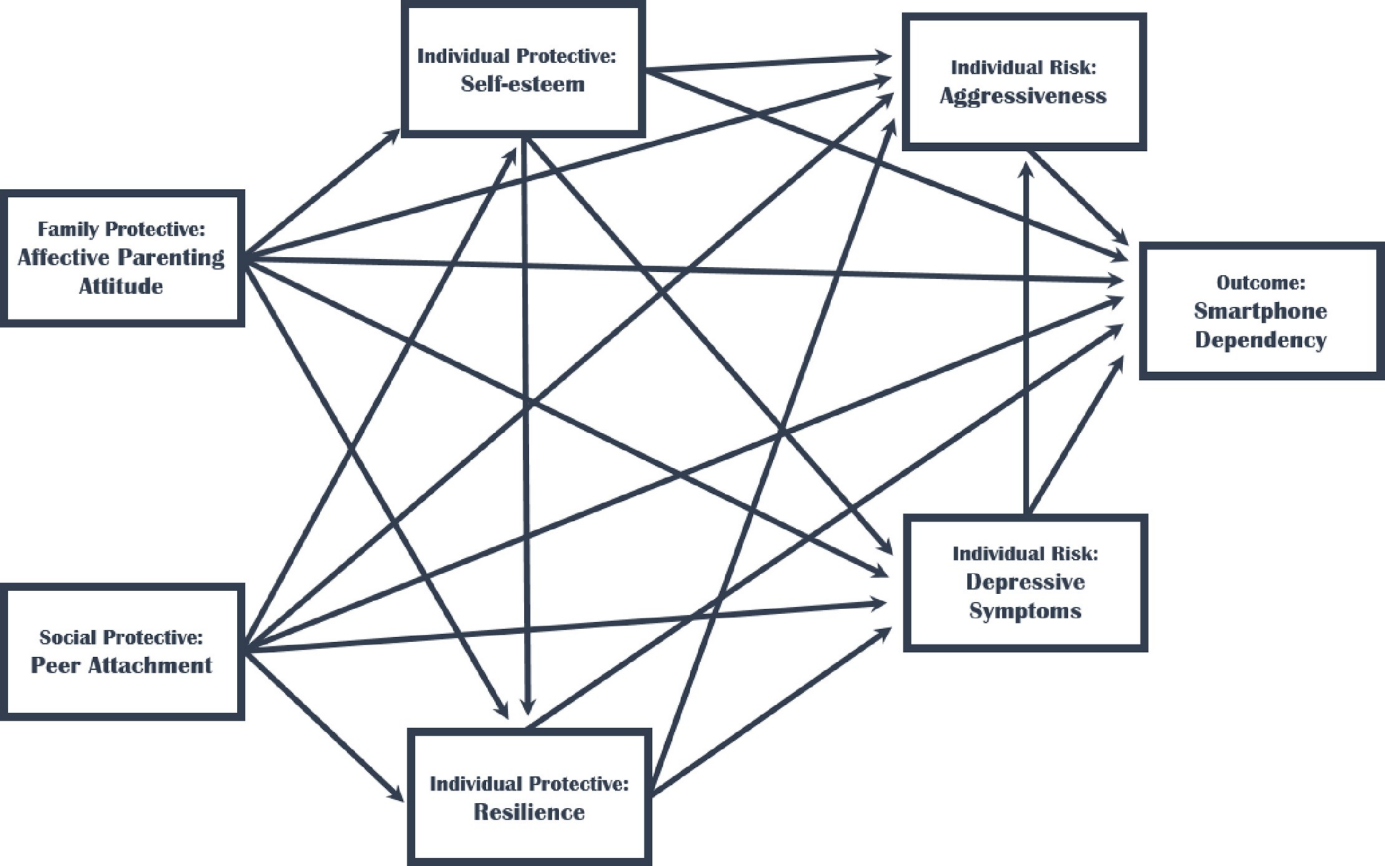
Hypothetical path model diagram.

We established a path model of adolescents’ smartphone dependency based on the Adolescent Resilience Model of Hasse [16] and a literature review (Fig 1). Consequently, we included affective parenting attitudes and peer attachment, which are social factors that affect smartphone dependency [12,13]. We included depressive symptoms and aggressiveness because they are individual risk factors associated with smartphone dependency [5,17], and included self-esteem and resilience as protective factors [15]. Additionally, affective parenting attitudes not only affect smartphone dependency, but also have a significant effect on adolescents’ psychological adaptation [11]. Further, peer groups and peer attachment have a psychosocial impact during adolescence [14,17]; therefore, we included affective parenting attitudes, peer attachment, self-esteem, resilience, depressive symptoms, and aggressiveness in our hypothesized model of smartphone dependency.

## Materials and methods

### Study design

This path analysis was conducted to identify the factors that affect smartphone dependency in adolescents and the direct and indirect paths between these factors by conducting a secondary analysis of the 7th panel investigation of 12-year-old adolescents in the 2016 Korean Children and Youth Panel Survey by the National Youth Policy Institute [18].

### Data source

We downloaded the data from the National Youth Policy Institute data archive homepage in April 2018. Data were collected from October to December 2016 through interviews with trained interviewers. Korean Children and Youth Panel surveys were sampled in a stratified multistage cluster sampling method for seven years from 2010 through 2016 over three school grades (1st, 4th, and 7th). The investigation was performed so that there were at least three schools from each city through the proportional allotment of 16 provinces and a sample class was randomly selected from each school. Among the 2,342 7th grade students sampled in 2016, 1,840 were included in the final analysis after excluding those with missing values and those who did not use smartphones.

### Ethical consideration

The National Youth Policy Institute provides the Korean Children and Youth Panel Survey to be used freely for academic purposes. All data from adolescents that completed the Korean Children and Youth Panel Survey were collected after obtaining parents’ written consent. The data used in this paper are public and anonymous; thus, informed consent and approval from an institutional review board were not required.

### Measures

#### Affective parenting attitude

For parenting attitude as perceived by adolescents, the Affective Parenting Attitude subscale was measured which comprised four items from Huh’s Parenting Behavior Inventory [19]. Each item was evaluated on a 4-point Likert scale, with higher total scores indicating more affective parenting attitudes. Cronbach’s α was .83 in this study.

#### Peer attachment

Peer attachment was measured using Armsden and Greenberg’s Inventory of Parent and Peer Attachment, which Hwang revised to exclude duplicated items by subscale [20]. This measure consists of nine items (communication, trust, and isolation, three items each). Each item is scored from 1 (*Almost Never or Never True)* to 4 (*Almost Always or Always True*). The three isolation items are reverse-coded, and higher total scores indicate higher levels of positive peer attachment. Cronbach’s α was .81 in this study.

#### Self-esteem

Self-esteem was measured using Rosenberg’s Self-Esteem Scale [21]. This is a 10-item, self-report questionnaire (positive and negative self-esteem; five items each). Each item is evaluated on a 4-point Likert scale, and the five negative self-esteem items are reverse-coded. Higher scores indicate increased positive perception regarding one’s value and competence. Cronbach’s α was .84 in this study.

#### Resilience

Resilience was measured using Block and Kremen’s Ego-Resilience scale that Yoo and Sim revised [22]. This scale consists of 14 items and is evaluated with a 4-point Likert scale. Higher total scores indicate higher resilience. Cronbach’s α was .88 in this study.

#### Depressive symptoms

Depressive symptoms were measured using 10 items from the revised 13-item Depression scale from the Symptom Checklist-90, which was validated into Korean after excluding three items [23]. It is evaluated with a 4-point Likert scale, and higher total scores indicate more severe levels of depressive symptoms. Cronbach’s α was .90 in this study.

#### Aggressiveness

Aggressiveness was measured using six items of a scale developed by Cho and Lim [24], including items such as “I get into fights over small things.” Aggressiveness was evaluated on a 4-point Likert scale and higher total scores indicate more aggressiveness. Cronbach’s α was .82 in this study.

#### Smartphone dependency

Smartphone dependency was measured using a seven-item instrument established in Lee and colleagues’ study [25]. The items related to dependence on mobile phone use, psychological dependence to mobile phone, and anxiety when terminating the use of mobile phones. Responses were rated using a 4-point Likert scale, and higher scores indicate higher smartphone dependency. Cronbach’s α was .90 in this study.

### Statistical analyses

Data were analyzed using SPSS/WIN 21.0(IBM, Armonk, NY, USA) and AMOS 23.0(SPSS, Chicago, IL USA). Skewness and kurtosis were examined to confirm that study variables were normally distributed, and significance was set at .05. Descriptive statistics included frequency, mean, standard deviation, and percentage, which were used to describe participants’ sociodemographic characteristics and study variables. Multicollinearity was evaluated using Pearson’s correlation coefficients among variables.

For the goodness-of-fit of the model, we used the χ^2^ statistic, Root Mean Square Error of Approximation, Comparative Fit Index, and Normed Fit Index complementarily to evaluate model fitness. To identify the direct and indirect effects of variables related to smartphone dependency, the measurement model and the structural model were analyzed. To estimate the fitness of the model and path coefficient and analyze the effects, we used the maximum-likelihood estimation method and a bootstrapping procedure.

## Results

### Participants’ sociodemographic characteristics

Participants mean age was 12.91±0.31 years, and there were an approximately even number of boys and girls. Most participants lived with both parents (94.4%), had siblings (88.9%), lived in urban areas (79.2%), attended coeducational schools (67.7%), and perceived themselves as having “middle” or “high” socioeconomic status (95.7%; Table 1)

**Table 1:** Participants’ sociodemographic characteristics (N = 1,840).

Variable	Category	n (%) or M±SD
**Age**		12.91±0.31
**Sex**	Boys	929 (50.5)
	Girls	911 (49.5)
**Perceived economic status**	High	708 (38.5)
	Middle	1052 (57.2)
	Low	80 (4.4)
**Family structure (i.e., live with)**	Both parents	1737 (94.4)
	One parent	93 (5.1)
	Grandparents	10 (0.5)
**Sibling(s)**	Yes	1636 (88.9)
	No	204 (11.1)
**Living area**	Urban	1458 (79.2)
	Rural	373 (20.3)
	Missing	9 (0.5)
**Nature of school**	All boys	290 (15.8)
	All girls	304 (16.5)
	Coeducational	1246 (67.7)

### Descriptive statistics and inter-correlation coefficient in measured variables

The correlation matrix among the measured variables and the descriptive statistics is are depicted in Table 2. Analysis revealed that the relationships among all variables were significant. Smartphone dependency was positively correlated with depressive symptoms and aggressiveness and negatively correlated with affective parenting attitude, peer attachment, self-esteem, and resilience.

**Table 2:** Correlation coefficients, means, and standard deviations of the study variables (N = 1840).

Variable	1	2	3	4	5	6	7	M ± SD	Range	Kurtosis	Skewness
	r	r	r	r	r	r	r				
1. Depressive symptoms	1	-.354***	-.427***	-.669***	-.403***	.615***	.344***	16.73 ± 5.49	10–40	0.425	0.738
2. Affective parenting attitude		1	.447***	.439***	.445***	-.278***	-.190***	13.16 ± 4.74	5–16	-0.121	-0.472
3. Peer attachment			1	.490***	.452***	-.290***	-.198***	28.48 ± 4.19	11–36	-0.054	0.041
4. Self-esteem				1	.489***	-.446***	-.340***	30.97 ± 4.76	12–40	-0.022	-0.337
5. Resilience					1	-.273***	-.202***	42.10 ± 6.65	14–56	-0.054	0.082
6. Aggressiveness						1	.380***	11.03 ± 3.42	6–24	-0.363	0.341
7. Smartphone dependency							1	15.19 ± 4.63	7–28	0.447	0.178

***p < .001. M = mean, SD = standard deviation

The skewness and kurtosis values of all variables did not exceed the absolute value of 2. Therefore, the problem of multicollinearity could be ruled out because the correlations among all independent variables did not exceed the criterion value of .70.

### Model fit

The path model was saturated (χ^2^ = .000) and the “0” degree of freedom did not place any restrictions on the parameter. It was a completely fitted model for this data. The χ^2^-value of .000 signifies that there was no difference between the covariance matrix obtained from the sample and the covariance matrix estimated from the model. The statistical value of χ^2^ is inversely proportional to *p-*value; thus, because the null hypothesis could not be rejected, this model can be said to be fit to the data. Furthermore, degrees of freedom refer to the difference between the number information and the number of unknown information that cannot be identified and is calculated by the total number of information minus the parameter. A negative (-) degree of freedom indicates under-differentiation, while a positive (+) degree of freedom indicates over-differentiation. The “0” degree of freedom in our model indicates a fitting differentiation. The research model that is completely fitting to the data like this is called a “saturated model,” which signifies that the model fit does not need to be evaluated [26].

### Parameter estimation and significance of the path model

**Fig 2:**
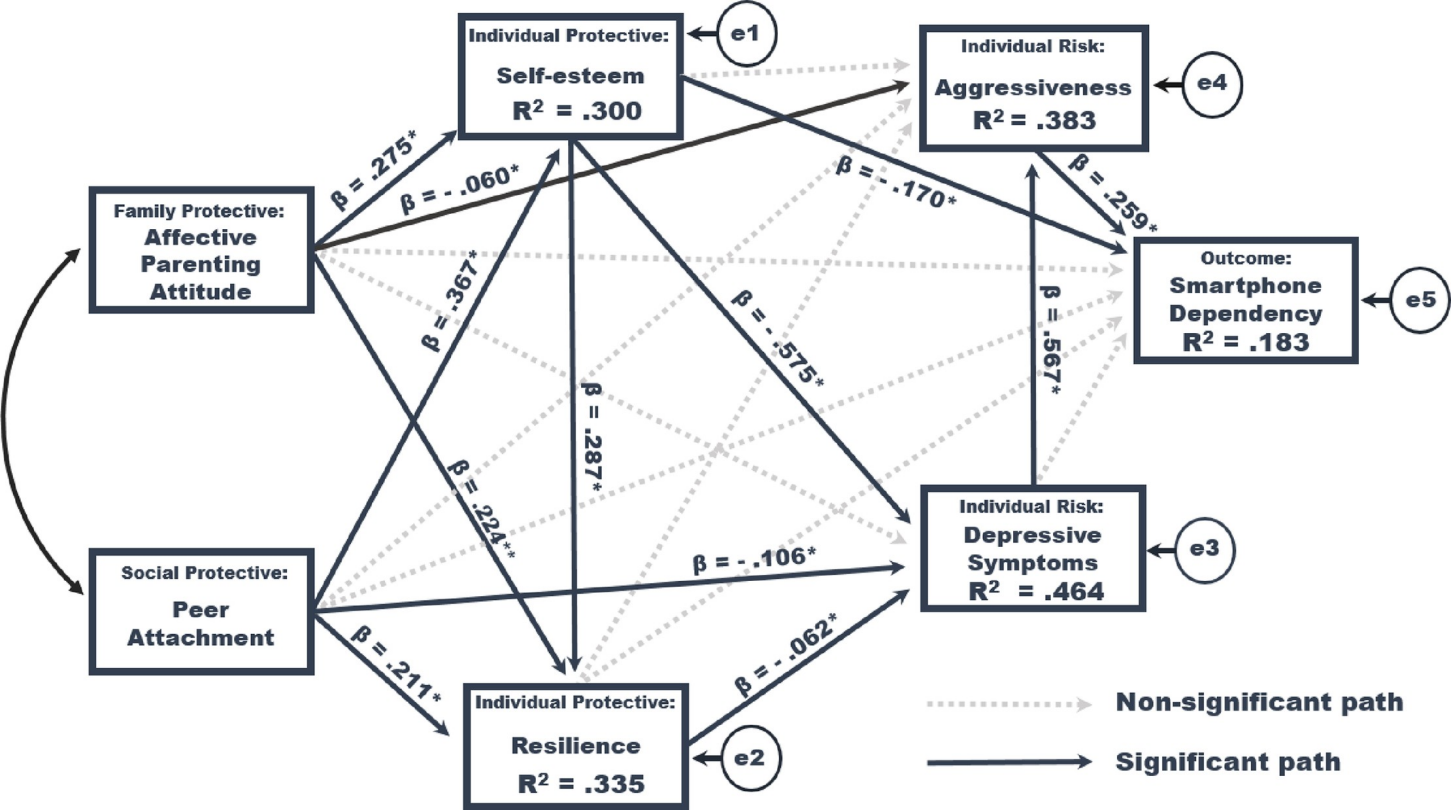
A path diagram of the study. Model fit statistics: χ^2^ = 0.00, *df* = 0. Solid lines represent significant standardized path coefficients (*p < .05, **p < .001). Error variances appear in small circles.

Fig 2 shows the path based on the parameters of the standardized path of the path model of our research. The paths that had a significant direct effect on self-esteem were parents’ affective parenting attitude as perceived by adolescents (β = .275, *p* = .001) and peer attachment (β = .367, *p* = .001), while the paths that had a significant direct effect on resilience were affective parenting attitude (β = .224, *p* < .001), peer attachment (β = .211, *p* = .001), and self-esteem (β = .287, *p* = .001). The paths that had a significant direct effect on depressive symptoms were peer attachment (β = -.106, *p* = .001), self-esteem (β = -.575, *p* = .001), and resilience (β = -.062, *p* = .015), while the paths that had a significant direct effect on aggressiveness were affective parenting attitude (β = -.060, *p* = .027) and depressive symptoms (β = .567, *p* = .001). The paths that had a significant direct effect on smartphone dependency were self-esteem (β = -.170, *p* = .001) and aggressiveness (β = .259, *p* = .001).

### Path model analysis

The results of implementing bootstrapping to analyze the direct, indirect, and total effects in the hypothesis path model are shown in Table 3. Affective parenting attitude and peer attachment had significant direct effects and explained 30% of self-esteem. The variables that had a significant direct effect on resilience were affective parenting attitude, peer attachment, and self-esteem; among those, affective parenting attitude and peer attachment had significant indirect effects mediated by self-esteem and explained 33.5% of resilience.

**Table 3:** Standardized direct, indirect, and total effects of study variables (N = 1840).

Endogenous variable	Exogenous variable	Direct effect	Indirect effect	Total effect	SMC
		β (*p*)	β (*p*)	β (*p*)	%
Self-esteem	← Affective parenting attitude	.275 (.001)	-	.275 (.001)	30.0
	← Peer attachment	.367 (.001)	-	.367 (.001)	
Resilience	← Affective parenting attitude	.224 (< .001)	.079 (< .001)	.303 (< .001)	33.5
	← Peer attachment	.211 (.001)	.105 (.001)	.317 (.001)	
	← Self-esteem	.287 (.001)	-	.287 (.001)	
Depressive symptoms	← Affective parenting attitude	-.026 (.281)	-.177 (.001)	-.203 (.001)	46.4
	← Peer attachment	-.106 (.001)	-.230 (.001)	-.337 (< .001)	
	← Self-esteem	-.575 (.001)	-.018 (.013)	-.592 (.001)	
	← Resilience	-.062 (.015)	-	-.062 (.015)	
Aggressiveness	← Affective parenting attitude	-.060 (.027)	-.125 (.001)	-.185 (.001)	38.3
	← Peer attachment	-.003 (.890)	-.205 (< .001)	-.208 (< .001)	
	← Self-esteem	-.040 (.206)	-.335 (.001)	-.375 (.001)	
	← Resilience	.003 (.907)	-.035 (.014)	-.033 (.316)	
	← Depressive symptoms	.567 (.001)	-	.567 (.001)	
Smartphone dependency	← Affective parenting attitude	-.014 (.592)	-.112 (.001)	-.126 (.001)	18.3
	← Peer attachment	.000 (.999)	-.142 (.001)	-.141 (< .001)	
	← Self-esteem	-.170 (.001)	-.137 (.001)	-.307 (.001)	
	← Resilience	-.019 (.508)	-.012 (.175)	-.031 (.275)	
	← Depressive symptoms	.059 (.129)	.147 (.001)	.206 (.001)	
	← Aggressiveness	.259 (.001)	-	.259 (.001)	

SMC = square multiple correlation

The variables that showed direct effects on depressive symptoms were peer attachment, self-esteem, and resilience, and the variables that showed a significant indirect effect were affective parenting attitude, peer attachment, and self-esteem, explaining 46.4% of depressive symptoms. The direct effect of affective parenting attitude on depressive symptoms was non-significant; however, the indirect effect shown by mediating self-esteem and resilience was significant.

The variables that showed a direct effect on aggressiveness were affective parenting attitude and depressive symptoms; and affective parenting attitude, peer attachment, self-esteem, and resilience showed significant indirect effects and explained 38.3% of the variance of the variable of aggressiveness.

The variables that showed significant direct effects on smartphone dependency were self-esteem and aggressiveness; and variables that showed indirect effects were affective parenting attitude, peer attachment, self-esteem, and depressive symptoms. These variables explained 18.3% of smartphone dependency.

## Discussion

This study comprehensively explored the factors that affect adolescents’ smartphone dependency by using data from the Korean Children and Youth Panel Survey. Our results revealed that self-esteem directly affected smartphone dependency and indirectly affected smartphone dependency by mediating the relationship between depressive symptoms and smartphone dependency, which confirms previous findings [15]. As adolescents with low self-esteem prefer communication through smartphones, texting, and social network services over face-to-face contact, they report difficulty with maintaining interpersonal relationships, which could lead to increased smartphone dependency [27].

In this study, aggressiveness also directly affected smartphone dependency. For adolescents who do not appropriately express various pressures and urges, the virtual world of the Internet becomes a space in which internal aggressiveness can be expressed, confirming prior research [5]. In addition, adolescents with higher aggressiveness experience difficulty forming peer relationships [28], and smartphone dependency worsens the ability to resolve negative emotions and conflicts experienced in daily life [9].

Various interventions could be used with aggressive adolescents. One study reported that a group music intervention was effective at increasing self-esteem and decreasing aggressiveness [29]. Therefore, to reduce adolescents’ smartphone dependency, promoting interventions that increase self-esteem and resolve aggressiveness may be effective.

In this study, the factors that had indirect effects on smartphone dependency were affective parenting attitude, peer attachment, self-esteem, and depressive symptoms, which emphasizes the importance of parental and peer relationships. Although affective parenting attitude and peer attachment did not have significant direct effects on smartphone dependency, they showed significant indirect effects by mediating self-esteem, depressive symptoms, and aggressiveness, which are key psychological factors. Twelve-year-old Korean youths, who were the subject of this study, have higher expectations and obligations for academic achievement than do their younger counterparts. This burden can seriously affect the psychological health of Korean adolescents [30]. In this period of psychological instability and vulnerability, relationships with parents and peers can have buffering effects, thus promoting adolescents’ healthy psychological state. This is similar to previous results that showed that parents’ affective attitude and peer attachment increased self-esteem and decreased aggressiveness among adolescents [31]. On the other hand, the smartphone penetration rate among Korean adolescents is the highest in the world [2], and we showed that this is associated with varied psychological problems. Therefore, interventions or policy decisions require a thorough understanding of adolescents’ social environment, which may affect their behavior, such as what parenting they are experiencing and what attachment relationships they have with their peer groups.

Depressive symptoms had a direct effect on smartphone dependency, which was consistent with prior studies that revealed both direct and indirect effects [5,15]. Adolescents with depression might use smartphones as a method to reduce or avoid depressive symptoms, which leads to increased dependency. Therefore, addressing psychological issues is necessary when establishing intervention and prevention strategies for adolescents’ smartphone dependency.

Resilience did not have a significant indirect or direct effect on dependency in the current study. This is similar to the results of a previous study that identified the factors that affect smartphone addiction in college students [32], which reported that resilience was not significantly related to smartphone addiction. However, this differs from another study of adolescents [15] that found resilience to be significantly related to smartphone addiction. Perhaps a sub-concept of resilience, such as self-control, is related to smartphone dependency and/or addiction [12]. However, in this study, the Korean translation of Block and Kremen’s Ego-Resilience scale was used to measure resilience, and this scale measures the emotional control that emphasizes other-related perspectives rather than behavioral control on oneself. Further in-depth research that addresses the relationship between resilience and smartphone dependency is needed in the future.

### Limitations

This study had some limitations. First, we used panel data consisting of self-report surveys, which cannot exclude researcher bias, and response-biases are possible. Therefore, future studies must consider methods to increase the validity of the results through direct observations or interviews related to smartphone use. Second, because as the age of first-time smartphone use is lowering, smartphone dependency research across various age groups is necessary. Finally, we used panel data, the research focused on the psychological and social factors that affect smartphone dependency; however, we failed to consider smartphone use time and the specific reasons for using smartphones. Therefore, future research should focus on a multifaceted and in-depth designs that includes physical health factors.

### Conclusions

This study is significant in that it tangibly identified the psychological and social factors that affect smartphone dependency by presenting direct and indirect paths among factors and establishing a hypothetical model. The main findings of this study are as follows. First, adolescents’ self-esteem and aggressiveness had significant direct effects on smartphone dependency. Second, affective parenting attitude, peer attachment, self-esteem, and depressive symptoms had significant indirect effects on smartphone dependency. Consequently, adolescents’ smartphone dependency is multifaceted and affected by a complex mixture of direct and indirect effects; therefore, when establishing methods to reduce adolescents’ smartphone dependency, these interactive relationships must be sufficiently considered.
